# Preclinical Evaluation of Bacterial Lysates in Murine Disease Models: A Systematic Review

**DOI:** 10.1002/iid3.70553

**Published:** 2026-10-15

**Authors:** Diana‐Larisa Ancuța, Cristin Coman

**Affiliations:** ^1^ Cantacuzino National Military‐Medical Institute for Research and Development Bucharest Romania; ^2^ Faculty of Veterinary Medicine University of Agronomic Sciences and Veterinary Medicine Bucharest Romania; ^3^ Center of Excellence in Translational Medicine Fundeni Clinical Institute Bucharest Romania

**Keywords:** bacterial lysates, disease models, mice, OM‐85, postbiotics, respiratory infections

## Abstract

**Background:**

Bacterial lysates are biologically active preparations obtained by thermal, mechanical, or chemical inactivation of whole bacterial cells, preserving their immunomodulatory properties.

**Methods:**

This systematic review evaluates preclinical studies published between 2010 and 2025 that investigated bacterial lysates in murine disease models, according to the PRISMA 2020 guideline. A total of 34 original studies met the inclusion criteria. The included studies cover a wide spectrum of pathologies, with respiratory infections and allergic/asthmatic diseases representing the most extensively investigated areas, followed by recurrent urinary tract infections, inflammatory bowel disease, acute lung injury/fibrosis, neuroinflammation, and cancer. Lysates ranged from monobacterial preparations to complex polybacterial formulations (particularly OM‐85), administered orally, intranasally, or systematically.

**Results:**

Key mechanisms include activation of TLRs and NOD receptors, modulation of the NLRP3 inflammasome, T‐cell polarization, macrophage remodeling, and induction of trained immunity. Beneficial effects were consistently reported, including reduced allergic inflammation, protection against recurrent infections, barrier restoration, and potentiation of anticancer immunotherapy.

**Conclusions:**

The most robust and translationally advanced evidence remains that obtained in respiratory infection models, the original clinical indication of bacterial lysates. Clinical translation is still limited by preparation heterogeneity, lack of standardization, differences between murine and human immunity, and predominance of preventive study designs. Methodological standardization and rigurous bridging studies are needed to realize the therapeutic potential of bacterial lysates.

**Trial Registration:**

PROSPERO (ID: CRD420261282071).

## 1 Introduction

Bacterial lysates are a heterogeneous group of biologically active preparations obtained by chemical, mechanical, or thermal disruption of whole bacterial cells. In contrast to live probiotics, bacterial lysates contain inactivated microbial components, such as cell wall fragments, nucleic acids, lipoproteins, and pathogen‐associated molecular patterns (PAMPs), which retain their potent immunomodulatory properties. Traditionally, bacterial lysates have been used for the prevention of recurrent respiratory tract infections, particularly in pediatric and elderly populations [1]. However, over the past 15 years, accumulating evidence from preclinical studies has significantly expanded the proposed therapeutic spectrum beyond the respiratory system [2]. From a mechanistic perspective, bacterial lysates exert their biological effects primarily through the activation of innate immune receptors, including Toll‐like receptors (TLRs), NOD‐like receptors (NLRs), and C‐type lectin receptors [1]. These interactions promote downstream signaling pathways that modulate antigen presentation, cytokine secretion, and adaptive immune polarization [3]. Importantly, such effects do not require the presence of viable bacteria, placing bacterial lysates in the emerging category of postbiotics—nonviable microbial products with demonstrated health benefits [4], as defined by the International Scientific Association for Probiotics and Prebiotics [5]. Murine models have emerged as the dominant experimental platform for investigating the systemic effects of bacterial lysates. This predominance is explained by several factors: (i) the high degree of genetic manipulability of mice, allowing for precise mechanistic dissection; (ii) the availability of standardized disease models that closely mimic human inflammatory, allergic, autoimmune, oncological, and infectious diseases; and (iii) the feasibility of controlled modulation of the microbiota [6]. Consequently, murine models have become indispensable for exploring both the therapeutic potential and the mechanistic underpinnings of bacterial lysates.

Between 2010 and 2025, the scope of preclinical research on bacterial lysates has expanded substantially. Beyond respiratory infections, researchers have explored their effects in experimental models of inflammatory bowel disease [6], atopic dermatitis, allergic asthma [7, 8], autoimmune encephalomyelitis, urinary tract infections, neuroinflammation, cancer, and chemotherapy‐induced immunosuppression [9]. These studies consistently suggest that bacterial lysates act as systemic immunomodulators, capable of remodeling both the innate and adaptive immune landscapes [10].

Despite these advances, several critical challenges remain. The diversity of bacterial lysate preparations, variability in dosing regimens, differences in administration routes, and lack of standardization between experimental protocols severely limit comparability between studies. Furthermore, the translational relevance of murine findings to human physiology remains a subject of active debate, especially in the context of the complexity of the immune system and microbiota‐host interactions [1, 11].

Therefore, this systematic review aims to comprehensively evaluate preclinical studies published between 2010 and 2025 that investigated bacterial lysates in murine disease models. Specifically, we aim to (i) classify the disease models explored, (ii) summarize the types of bacterial lysates and the administration strategies used, (iii) analyze the reported immunological mechanisms, (iv) evaluate safety and efficacy signals, and (v) critically discuss the translational potential and current limitations of this rapidly evolving field.

## 2 Systematic Review Methodology

This systematic review was conducted in accordance with the PRISMA 2020 (Preferred Reporting Items for Systematic Reviews and Meta‐Analyses) guideline to ensure methodological rigor, transparency, and reproducibility. The review protocol was registered in PROSPERO, the International Prospective Register of Systematic Reviews (ID: CRD420261282071), before data extraction began. In preparation for the review, pilot tests were conducted to optimize the search strategy. Subsequently, a formal search of studies was performed in the identified databases, and the results were assessed according to predefined inclusion and exclusion criteria. This approach allowed for a systematic and transparent selection of included studies and ensured the coherence and reproducibility of the methodological process.

### 2.1 Literature Search Strategy

A comprehensive search of the scientific literature was performed using the following electronic databases: PubMed/MEDLINE, Scopus, and Web of Science Core Collection. The search covered articles published between January 1, 2010 and March 31, 2025, with no restrictions on the country of origin of the studies.

The search strategy was based on combinations of controlled vocabulary (MeSH terms) and free‐text keywords, including: “bacterial lysate,” “heat‐inactivated bacteria,” “tyndallized bacteria,” “postbiotic,” “murine model,” “mouse,” “in vivo,” “immunomodulation,” and “experimental disease.” The reference lists of all eligible original articles and relevant review papers were also manually reviewed to identify additional studies.

### 2.2 Eligibility Criteria

Studies were included based on the following predefined criteria. Inclusion criteria were original in vivo experimental studies using murine models (any strain, sex, or age) with the administration of bacterial lysates, heat‐inactivated bacteria, or tyndallized bacterial preparations, evaluated in any disease model or physiological condition, published between 2010 and 2025, and available in English.

Exclusion criteria included in vitro studies without in vivo validation, studies in animal models other than mice, human clinical trials, studies using live probiotics exclusively, vaccination studies using purified antigens without the lysate structure, and conference abstracts, editorials, and reports that had not undergone peer review.

Studies that reported both murine and human data were included only if the experimental component involving mice could be extracted and analyzed independently. Studies in which the murine data could not be clearly separated from the human data were excluded in order to maintain a strict focus on preclinical murine evidence.

### 2.3 Study Selection Process

Two independent reviewers screened titles and abstracts for relevance. Full‐text articles of potentially eligible studies were then assessed for final inclusion. Disagreements were resolved by consensus or by consultation with a third reviewer.

The study selection process consisted of three sequential phases: identification of records by database search, screening based on titles and abstracts, and assessment of eligibility by full‐text review. A PRISMA flow chart was constructed to illustrate the selection process (Figure 1).

**Figure 1:**
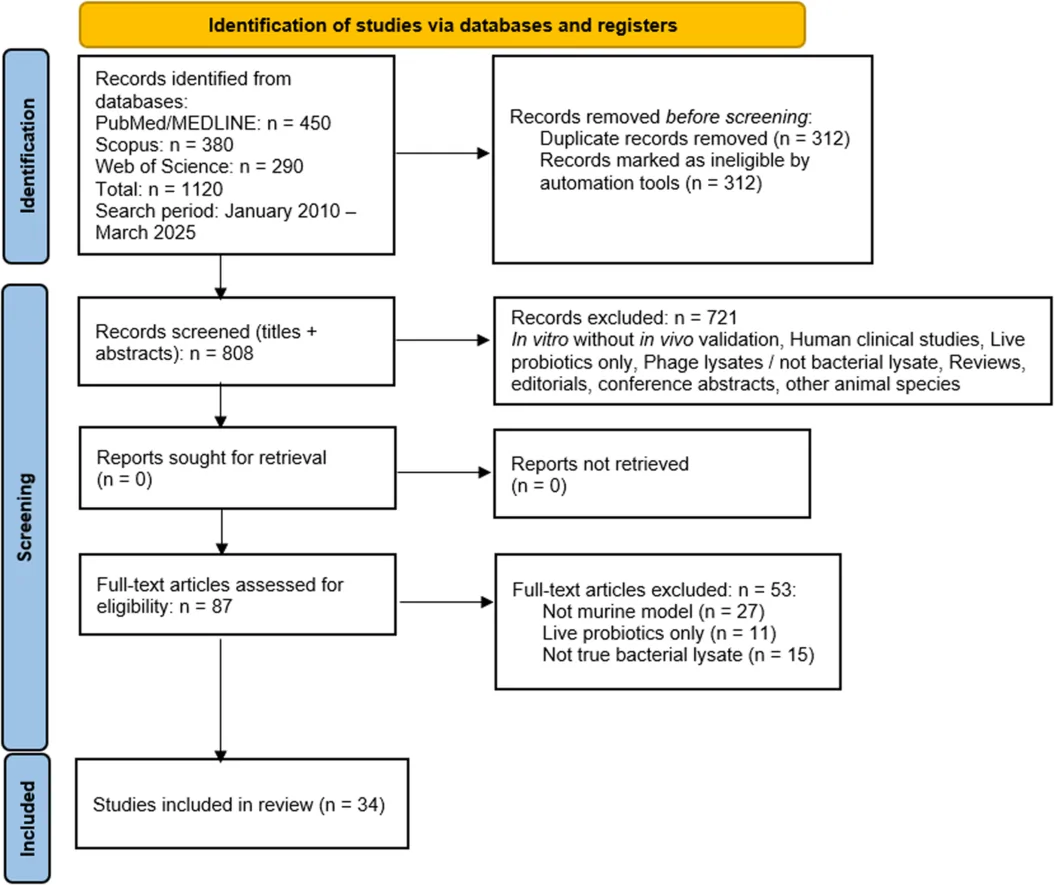
PRISMA flow diagram of the systematic literature search and study selection process for preclinical evaluation of bacterial lysates in murine disease models. The diagram was created using the official PRISMA 2020 template [12], available under the Creative Commons Attribution license (CC BY 4.0).

### 2.4 Data Extraction

From each included study, the following data were systematically extracted: first author and year of publication, murine strain, age and sex of animals, type and origin of bacterial lysate, method of bacterial inactivation (mechanical, thermal, chemical), disease model, route of administration (oral, intranasal, aerosol, systemic), dosing regimen and duration of treatment, preventive versus therapeutic experimental design, immunological and clinical parameters evaluated, as well as main results and conclusions.

### 2.5 Data Synthesis

Given the marked heterogeneity of bacterial lysate formulations, dosing protocols, and disease models, a quantitative meta‐analysis was not performed. Instead, the results were synthesized using a structured narrative approach, with studies grouped by disease category, lysate type, route of administration, and underlying immunological mechanism.

A total of 34 original preclinical studies using mouse models met the inclusion criteria and were selected for analysis. The complete list of studies, with details on the mouse strain, type of lysate, disease model, route of administration, and main results, is presented in Table S1.

The eligible studies included 10 (29.4%) that investigated asthma/allergy patterns, 8 (23.5%) that investigated respiratory infections, 5 (14.7%) urinary tract infections, 4 (11.8%) inflammatory bowel disease/colitis, 3 (8.8%) acute lung injury or fibrosis, 2 (5.9%) metabolic or other conditions, 1 (2.9%) neuroinflammation (EAE), and 1 (2.9%) cancer. Standardized commercial lysates (particularly OM‐85) were used in 19 studies (55.9%), while research‐grade or laboratory‐prepared samples were used in 15 studies (44.1%). Oral administration (including via gastric tube and maternal route) was the most common, followed by the intranasal/aerosol route, which was used primarily in respiratory and asthma models.

### 2.6 Methodological Reporting Considerations

An overview of key methodological reporting elements across the included murine studies was performed using selected domains adapted from the SYRCLE framework, with the aim of contextualizing the preclinical evidence base rather than excluding studies. The assessment focused on transparency of reporting and did not identify major sources of systematic bias (Table 1).

**Table 1:** Overview of methodological reporting elements in the included murine studies (adapted from the SYRCLE framework).

Methodological reporting domain	Reporting pattern in the reviewed literature	Interpretative comment
Randomization of animals	Variable reporting	Randomization was not consistently described, reflecting common reporting practices in preclinical research
Allocation concealment	Rarely reported	Insufficient information for formal assessment; absence of reporting does not imply absence of the procedure
Blinding of investigators	Limited reporting	Blinding is not routinely implemented in exploratory murine studies
Blinding of outcome assessment	Variable reporting	More frequently reported for histological analyses
Handling of incomplete data	Adequately reported	Animal attrition was minimal and generally explained
Outcome reporting	Largely consistent	Reported outcomes were generally aligned with stated objectives
Other methodological considerations	No major concerns identified	No clear evidence of systematic bias across studies

## 3 Why the Mouse Is the Dominant Experimental Model for Bacterial Lysate Research

Murine models are the cornerstone of preclinical research on bacterial lysates, accounting for the vast majority of in vivo studies. This predominance is driven by a unique combination of genetic, immunological, and practical advantages that make the mouse an unparalleled experimental platform for studying host‐microbe interactions and immunomodulatory interventions.

First, the murine immune system has a high degree of structural and functional conservation with the human immune system. Key components of innate immunity—including TLRs, NLRs, inflammasome complexes, and subsets of antigen‐presenting cells—are highly homologous between mice and humans. This similarity is particularly relevant for bacterial lysates, whose primary mechanism of action involves pattern recognition receptor (PRR) signaling and downstream cytokine cascades. Therefore, murine models allow for mechanistic exploration of TLR2, TLR4, NOD2, and MyD88‐dependent pathways that are directly relevant to human immunobiology [13, 14, 15].

Second, genetic manipulation is a decisive advantage of murine systems. A wide range of transgenic, knockout, and reporter mouse lines is available, allowing for precise dissection of signaling pathways involved in lysate‐induced immune modulation. For example, studies using TLR‐, NLRP3‐, or IL‐10‐deficient mice have provided direct evidence that bacterial lysates modulate inflammasome activation, regulatory T cell differentiation, and innate immune memory. Such mechanistic resolution is rarely achievable in larger animal models [16, 17].

Third, mice provide access to a comprehensive repertoire of standardized disease models that closely recapitulate critical aspects of human pathology. These include chemically induced colitis (dextran sulfate sodium, DSS; trinitrobenzene sulfonic acid, TNBS), experimental autoimmune encephalomyelitis (EAE) as a model of multiple sclerosis, allergic asthma induced by ovalbumin or house dust mites, imiquimod‐induced dermatitis, syngeneic tumor implantation models, and models of controlled bacterial or viral infections. These platforms allow for consistent evaluation of bacterial lysates in diverse pathological contexts. Furthermore, the reproducibility of these models is facilitated by the homogeneous genetic background of inbred lines and standardized rearing and environmental conditions (diet, housing, microbiota), which reduces experimental variability and allows replication of results between laboratories [13, 18]. From a logistical and ethical perspective, mice offer significant advantages in terms of housing requirements, breeding efficiency, cost, and regulatory approval. Their short life cycle allows for rapid assessment of both short‐ and long‐term immunological effects, including developmental immune imprinting, trained immunity, and tolerance induction. Furthermore, the widespread availability of immunological reagents, monoclonal antibodies, and high‐throughput molecular tools further strengthens the translational value of murine research [13, 16].

However, the dominance of murine models also introduces inherent limitations. There are fundamental differences between murine and human microbiota composition, immune system maturation, cytokine expression profiles, and lifespan‐associated immune remodeling. Mice exhibit a higher basal metabolic rate, distinct neutrophil‐to‐lymphocyte ratios, and altered sensitivity to endotoxins compared with humans. These differences may profoundly influence the pharmacodynamics and systemic effects of bacterial lysates [15, 19].

It is important to note that most murine studies use young, healthy animals raised in specific pathogen‐free conditions, which do not adequately reflect the immunological history, environmental exposures, or comorbidities of human patients. This discrepancy raises concerns about the external validity and translational predictability of murine data, particularly for chronic inflammatory and autoimmune diseases [14, 20, 21]. Thus, mice constitute an indispensable experimental system for investigating immunological mechanisms and the therapeutic potential of bacterial lysates. Their unparalleled genetic tractability, standardized disease models, and methodological versatility have propelled major advances in the field. However, the translational limitations inherent in murine biology underscore the urgent need for complementary studies in higher‐order animal models and systems relevant to humans.

## 4 Types of Bacterial Lysates Used in Murine Studies

Murine studies investigating bacterial lysates have revealed substantial heterogeneity in terms of bacterial species composition, inactivation methods, and formulation strategies. This diversity reflects both the broad biological rationale underlying lysate‐based immunomodulation and the current lack of standardization in the field (Table 2).

**Table 2:** Types of bacterial lysates used in murine studies and key characteristics.

Lysate type and number of studies	Bacterial species	Inactivation method	Route of administration	Disease models investigated	Dominant immunological mechanisms
Commercial polybacterial (OM‐85) (approx. 19)	Eight species (*H. influenzae, S. pneumoniae, Klebsiella, Staphylococcus, Streptococcus, M. catarrhalis*, etc.)	Thermal/alkaline lysis	Oral, intranasal	Respiratory infections, asthma, acute respiratory distress syndrome (ARDS), fibrosis, obesity	Multi‐TLR activation, trained immunity, Tregs, reduction of IL‐33/TSLP
Research‐grade polybacterial (approx. 4–5)	Mixtures of *E. coli* + *Klebsiella, Proteus, Enterococcus, Staphylococcus*, etc. (e.g., UNAM‐HIMFG)	Thermal/mechanical/chemical	Oral, sublingual	Urinary tract infections (including recurrent)	Mucosal IgA induction, balanced cytokine response, bacterial clearance
Monobacterial (approx. 10–11)	*Lactobacillus casei, L. acidophilus, L. petauri, Lactococcus lactis, Parabacteroides distasonis, Bifidobacterium longum*, etc.	Thermal (heat‐killed)/mechanical	Oral	DSS colitis, EAE, colorectal cancer, allergy	Treg induction, barrier reinforcement, M1/M2 polarization, modulation of the microbiota

### 4.1 Polybacterial Lysates

Polybacterial lysates are complex mixtures derived from multiple bacterial strains, typically including both Gram‐positive and Gram‐negative species. These preparations are designed to maximize immune receptor engagement by exposing a broad range of PAMPs. In murine studies, polybacterial lysates have been used primarily in models of respiratory infections, but have been increasingly extended to models of systemic diseases, including autoimmune and inflammatory conditions, through strategic adaptations. For example, a recent study demonstrated that a heat‐killed multistrain “postbiotic” formulation conferred durable protection against recurrent urinary tract infections in mice [22]. Mechanistically, polybacterial lysates activate multiple TLRs (TLR2, TLR4, TLR5, and TLR9) as well as the NOD2 signaling pathways. This multivalent activation is thought to promote balanced immune stimulation while reducing the risk of excessive polarization toward a single T‐helper axis.

However, due to their complex and often proprietary composition, polybacterial lysates are difficult to characterize chemically and biologically, limiting reproducibility and comparability between studies.

### 4.2 Monobacterial Lysates

A substantial proportion of murine studies have used monobacterial lysates derived from individual bacterial strains, allowing for more precise mechanistic interpretation. The most commonly studied genera include: *Lactobacillus rhamnosus, Lactobacillus reuteri, Lactobacillus plantarum, Enterococcus* spp., *Parabacteroides* spp., *Escherichia coli*.

Monobacterial lysates offer several experimental advantages, including defined molecular composition, reduced batch‐to‐batch variability, and facilitated correlation between specific bacterial components and immunological outcomes. These preparations have been widely applied in models of inflammatory bowel disease, atopic dermatitis, neuroinflammation, and cancer. In particular, *Lactobacillus*‐derived lysates dominate the literature in studies of the gut–immune and gut–brain axis, where they have demonstrated consistent anti‐inflammatory and barrier‐protective effects [23].

### 4.3 Bacterial Inactivation Methods

Three main methods of bacterial inactivation have been used in murine studies.

#### 4.3.1 Thermal Inactivation (Tyndalized)

This is the most widely used approach and involves exposure to repeated thermal cycles. Thermal inactivation preserves cell wall structures and many immunostimulatory components while eliminating replicative capacity [22, 23].

#### 4.3.2 Mechanical Disruption (Sonication, High‐Pressure Homogenization)

This technique results in complete cell fragmentation, releasing intracellular nucleic acids and cytosolic proteins, in addition to membrane components [24, 25].

#### 4.3.3 Chemical Lysis, Rarely Used in Murine Models, due to the Risk of Degradation of Bioactive Components or Generation of Artifactual Compounds; Recent Literature Generally Avoids This Approach for Immunological Studies

Studies directly comparing live bacteria with heat‐inactivated counterparts indicate that many immunomodulatory effects—particularly those related to innate immune activation and barrier strengthening—are retained after inactivation, thus reinforcing the conceptual positioning of bacterial lysates as postbiotics [25, 26].

### 4.4 Classification of Postbiotics and Functional Implications

In the modern framework of microbiome‐based therapies, bacterial lysates are increasingly classified within the broader category of postbiotics, defined as nonliving microorganisms and/or their components that confer health benefits to the host. This classification reflects a paradigm shift away from the exclusive reliance on viable microbes and highlights the translational advantages of increased safety, stability, and regulatory compliance.

From a pharmacological perspective, lysates function as complex biological response modifiers, capable of stimulating mucosal immunity, inducing trained innate immunity, promoting regulatory immune pathways, and remodeling host–microbe dialogue without the risks associated with bacterial translocation or horizontal gene transfer [25].

### 4.5 Standardized Commercial Lysates Versus Research‐Grade Preparations

An important distinction in the reviewed literature is between standardized commercial lysates and laboratory‐prepared (research‐grade) preparations. Commercial lysates, particularly OM‐85 (Broncho‐Vaxom), are produced according to controlled manufacturing processes, with batch‐to‐batch standardization and documented clinical use. These were used in 19 of the 34 included studies (Table S1). In contrast, research‐grade preparations are produced by research groups, often from single strains (monobacterial) or locally defined polybacterial mixtures, and exhibit greater variability in terms of inactivation method, dose, and molecular characterization. Although research‐grade preparations allow for a more precise mechanistic interpretation, the lack of standardization limits comparability and translational potential. This methodological difference represents a major obstacle to reproducibility and to the transition from murine models to clinical studies.

### 4.6 Lack of Standardization as a Critical Limitation

Despite their broad applicability, bacterial lysates suffer from a major limitation: the lack of standardized production protocols, quality control parameters, and equivalence of dosing across studies. Even when using the same bacterial species, variations in culture conditions, inactivation temperature, treatment duration, and formulation matrix can profoundly alter the final immunological profile of the lysate.

This lack of standardization represents one of the most significant obstacles to translational reproducibility and underscores the urgent need for harmonized guidelines in preclinical lysate research.

## 5 Disease Models Investigated in Murine Studies

### 5.1 Models of Respiratory Infections

Respiratory infections represent the classic clinical indication and the most advanced one from a translational perspective for bacterial lysates. Eight studies have evaluated the effects of these preparations in murine models of viral infections (influenza, respiratory syncytial virus [RSV], murine coronavirus) or bacterial infections (pneumococcal pneumonia), as well as in cases of bacterial superinfection following a viral infection [27, 28, 29, 30, 31, 32, 33, 34].

Oral administration of OM‐85 demonstrated protection against influenza virus infection and reduced susceptibility to superinfection with *Streptococcus pneumoniae*, improving survival and reducing signs of morbidity [27, 28]. Intranasal administration of the same lysate provided protection against RSV by inhibiting viral replication and reducing peribronchial and perivascular inflammation [30]. A polyvalent lysate prepared mechanically or chemically, administered as a single intranasal dose, protected mice against lethal pneumococcal pneumonia via a MyD88‐dependent mechanism independent of neutrophils, IL‐17A, or caspase‐1 activation [29].

Furthermore, OM‐85 demonstrated the ability to reduce the expression of Ace2 and Tmprss2 receptors in the murine lung (MyD88/Trif‐dependent) [31] and to confer protection by priming the innate immune system in models of murine coronavirus infection [34]. Maternal administration of OM‐85 during gestation also improved the offspring's resistance to neonatal respiratory infections [33].

These data confirm the ability of bacterial lysates to induce both antiviral and antibacterial protection in the respiratory tract, supporting their role as preventive immunomodulatory agents in respiratory tract infections.

### 5.2 Models of Allergic and Atopic Diseases (Asthma, Allergic Rhinitis)

The largest number of studies (*n* = 10) investigated the effects of bacterial lysates in murine models of allergic asthma and allergic rhinitis [7, 35, 36, 37, 38, 39, 40, 41, 42, 43].

Oral or intranasal administration of the standardized bacterial lysate OM‐85 consistently reduced airway eosinophilia, serum IgE levels, and Th2‐associated cytokines (IL‐4, IL‐5, IL‐13), while promoting the expansion of regulatory T cells (Tregs) and suppressing epithelial‐derived alarmins, particularly IL‐33 and TSLP [7, 35, 38]. Mechanistic studies further demonstrated that OM‐85 modulates the epithelial–IL‐33–ILC2 axis and reprograms dendritic cells (DCs) toward a tolerogenic phenotype, effects that were maintained even in germ‐free mice, indicating that microbiota‐independent mechanisms also contribute to its immunomodulatory activity [37, 40].

Maternal administration of OM‐85 during gestation and/or lactation reduced allergic inflammation in offspring through modulation of the IL‐33/ILC2 pathway and short‐chain fatty acid production [42]. In allergic rhinitis models, prolonged OM‐85 pretreatment attenuated nasal symptoms, eosinophilic infiltration, and Th2 cytokine production [36].

Similar protective effects were observed with heat‐killed monobacterial lysates of Bifidobacterium longum BBMN68 and *Lactococcus lactis*, which reduced allergic airway inflammation and restored immune homeostasis [39, 43]. Although the findings consistently support the anti‐allergic potential of bacterial lysates, differences in sensitization protocols, allergen exposure, dosing regimens, and routes of administration limit direct comparisons between studies.

### 5.3 Models of Inflammatory Bowel Disease/Colitis

Four studies evaluated bacterial lysates in DSS‐induced colitis models [22, 44, 45, 46]. Oral administration of *Lactobacillus casei* DN‐114 001 lysate alleviated disease severity in BALB/c mice, but not in SCID mice, indicating that its protective effect depends on an intact immune system. The beneficial effects were associated with restoration of intestinal barrier integrity through increased tight junction protein expression and reduced intestinal permeability [44]. Similarly, the commercial polybacterial lysate Coliphagein improved clinical and histological scores, increased fecal IgA levels, and reduced pro‐inflammatory chemokines, supporting its role in restoring intestinal homeostasis [47]. Heat‐killed lysates of *Lactococcus petauri*, *Lactococcus lactis* L8, and *Lacticaseibacillus paracasei* 6235 further reduced colonic inflammation, decreased tumor necrosis factor alpha (TNF‐α) expression, and favorably modulated the gut microbiota, with some studies reporting efficacy comparable to or greater than that of the corresponding live probiotics [22, 45, 46, 47]. Collectively, these findings suggest that bacterial lysates ameliorate experimental colitis primarily by restoring epithelial barrier function and modulating mucosal immune responses.

### 5.4 Urinary Tract Infections (UTIs)

Five studies investigated polyvalent bacterial lysates or postbiotic formulations in murine models of urinary tract infection, including recurrent infections and those caused by multidrug‐resistant pathogens [22, 23, 48, 49]. Sublingual administration of the polyvalent UNAM‐HIMFG lysate accelerated bacteriuria clearance, prevented chronic bladder inflammation, and promoted a balanced immune response characterized by coordinated TNF‐α and IL‐10 production [48, 49]. Similarly, multistrain heat‐killed postbiotic formulations administered orally reduced bladder and renal bacterial colonization, limited tissue inflammation, and provided sustained protection against both primary and recurrent infections, maintaining high rates of urinary sterility even after treatment discontinuation [22, 23]. Collectively, these findings suggest that bacterial lysates enhance pathogen clearance while preventing excessive inflammation, supporting their potential as alternatives or adjuncts to antibiotic therapy. However, methodological heterogeneity, including differences in bacterial composition, treatment schedules, administration routes, and outcome measures, together with the limited characterization of local urinary mucosal immunity, currently limits direct comparison between studies and highlights the need for standardized preclinical protocols.

### 5.5 Neuroinflammation and Autoimmune Diseases (EAE)

A recent study evaluated the immunomodulatory potential of a *Parabacteroides distasonis* lysate in the EAE model [50]. Preventive oral administration delayed disease onset and reduced clinical severity through expansion of regulatory T cells in the mesenteric and inguinal lymph nodes, remodeling of the gut microbiota by increasing beneficial genera and reducing segmented filamentous bacteria, and the absence of systemic translocation of bacterial antigens. These findings suggest that the lysate acts primarily by restoring immune tolerance through modulation of the gut–immune axis rather than direct systemic exposure. Nevertheless, the available evidence is limited to a single preventive study, and further work is required to determine its therapeutic efficacy after disease onset and to clarify its effects within the central nervous system.

### 5.6 Cancer and the Enhancement of Immunotherapy

One study investigated the combination of *Lactobacillus acidophilus* lysate with anti‐CTLA‐4 antibodies in the AOM/DSS murine colorectal cancer model [51]. Combination therapy significantly reduced tumor incidence compared with either treatment alone and promoted an antitumor tumor microenvironment characterized by increased effector CD8+ T cells, reduced regulatory T cells and M2 macrophages, and partial restoration of gut microbial homeostasis. Complementary analyses suggested that the lysate inhibited M2 macrophage polarization and enhanced antitumor immune responses, supporting its potential as an immunotherapeutic adjuvant. However, because bacterial lysates were evaluated only in combination with checkpoint blockade, their independent antitumor efficacy remains unclear, warranting further investigation.

### 5.7 Other Models (Acute Lung Injury, Fibrosis, Obesity)

Three studies evaluated bacterial lysates in LPS‐induced acute lung injury and bleomycin‐induced pulmonary fibrosis models [52, 53, 54]. OM‐85 attenuated pulmonary inflammation and reduced fibrosis by modulating the IFN‐γ/IL‐4 balance and Notch signaling. Additional studies demonstrated that OM‐85 alleviated high‐fat diet‐induced obesity, insulin resistance, gut dysbiosis, and nonalcoholic steatohepatitis [55], while heat‐killed *Lactobacillus* preparations accelerated experimental skin wound healing [26]. Although these findings broaden the potential therapeutic applications of bacterial lysates beyond infectious and immune‐mediated diseases, evidence remains limited by the small number of available studies and the diversity of experimental models.

## 6 Routes of Administration and Biological Implications of Bacterial Lysates: A Translational Perspective

The route of administration is a determining factor in the pharmacokinetics, immunomodulatory potential, and systemic effects of bacterial lysates in experimental murine models. Published research has systematically explored multiple routes of administration, each with distinct mechanistic implications and different efficacy profiles. A thorough understanding of these differences is essential for optimizing the design of preclinical studies and for efficient translation to clinical applications.

### 6.1 Oral Administration: Modulation of Intestinal Mucosal Immunity

Oral administration is the most commonly used route of administration for bacterial lysates, especially in experimental models of gastrointestinal diseases, systemic immunity, and neuroinflammation. The popularity of this route is due to both its noninvasive nature and its ability to extensively engage the immune system associated with the gastrointestinal tract.

From a mechanistic perspective, oral administration allows direct interaction of lysates with gut‐associated lymphoid tissue (GALT), including specialized anatomical structures such as Peyer's patches and mesenteric lymph nodes. This interaction triggers a complex cascade of immunological events: stimulation of DCs that function as sentinels of mucosal immunity, promotion of antigen presentation to naive T lymphocytes, activation and expansion of regulatory T lymphocytes (Tregs) that play a role in maintaining immunological tolerance, and fine‐tuning the balance between Th1, Th2, and Th17 subpopulations, leading to the orientation of the immune response toward balanced anti‐inflammatory or pro‐inflammatory profiles [56, 57].

Studies in diverse experimental models (DSS‐induced colitis, allergic asthma, and EAE) have convergently demonstrated that orally administered lysates can restore compromised intestinal barrier integrity by strengthening intercellular tight junctions, stimulate the production of secretory immunoglobulin A (sIgA), the dominant antibody of mucosal immunity that neutralizes pathogens at the luminal level, and influence systemic immunity without producing detectable bacteremia, a crucial aspect for biological safety [56, 58, 59].

A particularly fascinating aspect of oral administration is its ability to engage the gut‐brain axis, a complex bidirectional communication network through which microbial components can indirectly influence central nervous system immune responses, neuroinflammatory processes, and even behavior. This influence is mediated by multiple mechanisms: production of microbial metabolites that cross the blood–brain barrier, signaling via the vagus nerve, and modulation of circulating cytokines that affect glial and neuronal function [57, 60].

The advantages of oral administration are multiple and significant: noninvasiveness increases acceptability and adherence to treatment, engagement of mucosal immunity provides protection at the entry points of most pathogens, and the potential for both local and systemic effects maximizes therapeutic versatility. However, there are important limitations that must be considered: partial or complete degradation by gastric acid and digestive enzymes may compromise the bioactivity of lysates, variable absorption dependent on formulation and individual gastrointestinal status may generate inconsistency in therapeutic response, and translation of doses from mice to humans requires careful consideration of differences in gastrointestinal physiology, mucosal surface area, and metabolism between species [61].

### 6.2 Intranasal or Aerosol Administration: Targeting Respiratory Immunity

Intranasal administration represents an elegant strategy for targeting the upper respiratory tract and nasal‐associated lymphoid tissue (NALT), inducing mucosal immunity that can extend to distant mucosal sites through a phenomenon known as the “common mucosal immune system”—the functional interconnection of all mucosal lymphoid tissues.

In experimental models of allergic asthma, intranasally administered lysates, especially the standardized preparation OM‐85, have demonstrated impressive therapeutic effects: significant reduction of airway eosinophilia, characteristic of allergic inflammation, modulation of the production of epithelial alarmins such as IL‐33 and TSLP (thymic stromal lymphopoietin), which orchestrate early allergic responses, and stimulation of regulatory T lymphocyte activity that suppresses exaggerated Th2 responses [35, 62].

Aerosolized lysates have been used to induce local “immune training,” demonstrating the existence of a sophisticated dialogue between respiratory and systemic immunity. This concept of “trained immunity”—the innate memory of the immune system—suggests that exposure to microbial components can epigenetically reprogram innate immune cells, conferring cross‐protection against different pathogens [63, 64].

Advantages of this route of administration include targeted mucosal stimulation that maximizes local efficacy while minimizing systemic exposure, and rapid onset of immune activation due to the extensive vascularized surface area and immediate accessibility of nasal lymphoid tissue. Limitations include the potential for local irritation with risk of erosion or chronic inflammation, difficulty in precisely controlling the administered dose, and limited systemic distribution compared to oral administration, which may reduce efficacy in pathologies requiring generalized immune modulation [65, 66].

### 6.3 Systemic Administration: Subcutaneous or Intraperitoneal Injection

Systemic administration by subcutaneous or intraperitoneal injection is less commonly used in bacterial lysate research, but offers the advantage of directly exposing central immune organs—the spleen and lymph nodes—to bacterial components, completely bypassing mucosal barriers and enzymatic degradation processes. In murine models of malignant tumors, systemic administration of lysates in combination with immune checkpoint inhibitors such as anti‐CTLA‐4 antibodies has been shown to enhance cytotoxic T‐cell infiltration into the tumor microenvironment and to remodel the tumor immunological landscape toward an antitumor profile. This synergistic effect suggests that direct systemic immune activation may enhance the efficacy of oncological immunotherapy [67, 68]. Systemic routes are also used in experimental models of sepsis or acute infection to assess the capacity for rapid immune priming, that is, the accelerated preparation of the immune system to respond effectively to imminent infectious challenges. These studies have revealed that systemic exposure to bacterial components can modulate the threshold for immune activation and accelerate effector responses [69].

The advantages of systemic administration include complete bypass of mucosal barriers, eliminating problems of degradation or variable absorption, and direct and rapid systemic immune activation, ideal for situations requiring prompt responses. The limitations are significant: increased risk of immune overstimulation with potential for severe adverse reactions including shock or macrophage activation syndrome, potential toxicity due to high systemic concentrations of inflammatory components, and limited physiological relevance for mucosal diseases where local immunity is paramount [70].

### 6.4 Biological Implications of Route of Administration Selection

The selection of route of administration fundamentally determines the local versus systemic nature of immune engagement, the cell types predominantly activated, and the potential for induction of trained immunity or immunological tolerance. Oral and intranasal routes preferentially modulate mucosal immunity with secondary systemic effects propagated by circulating lymphocytes and soluble mediators, making them ideal for pathologies at the mucosal‐external interface. In contrast, systemic routes directly target peripheral immune compartments, providing rapid and intense control of the immune response, but with a potentially more problematic safety profile [65, 71].

Coordinated optimization of the route of administration, dose, and treatment frequency is essential to maximize therapeutic efficacy and minimize adverse effects in preclinical studies. This optimization must take into account the nature of the target pathology, the dominant immunopathological mechanisms, and the specific therapeutic goals—acute protection versus long‐term tolerance, local versus systemic modulation, immune stimulation versus immunosuppression. A deep understanding of these complex relationships is fundamental for the efficient translation of preclinical observations into clinical applications with real therapeutic impact [71, 72].

## 7 Immunological Mechanisms of Bacterial Lysates: A Complex Orchestration of the Immune Response

Bacterial lysates exert a broad spectrum of immunomodulatory effects in experimental murine models, engaging both innate and adaptive immune pathways in a coordinated and context‐dependent manner. The mechanisms by which these preparations influence the immune system are multifactorial and depend on a series of complex variables: the bacterial species from which the lysate is derived, the route of administration used, the disease model investigated, and the underlying immunological status of the host. A detailed understanding of these mechanisms is fundamental for optimizing therapeutic applications and predicting efficacy in different pathological contexts.

The mechanistic differences based on the route of administration are shown schematically in Figure 2.

**Figure 2:**
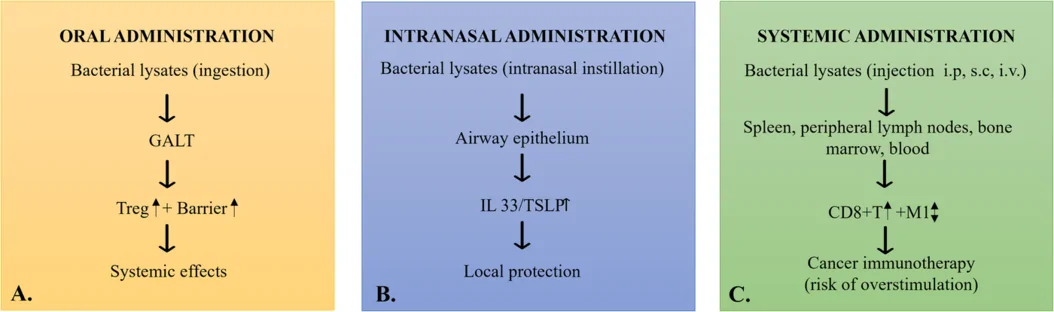
Immunological mechanisms of bacterial lysates categorized by route of administration. (A) Oral administration acts on gut‐associated lymphoid tissue (GALT), promotes the expansion of regulatory T cells (Tregs), and strengthens the epithelial barrier, generating systemic effects. (B) Intranasal administration targets the respiratory epithelium, reduces epithelial alarmins (IL‐33 and TSLP), and provides local protection in the airways. (C) Systemic administration strongly activates CD8+ T cells and M1‐type macrophages in the spleen and lymph nodes, which is useful for enhancing antitumor immunotherapy but is associated with the risk of immune overstimulation (figure created by the authors).

### 7.1 Pattern Recognition Receptors (PRRs): Sensory Gateways of Innate Immunity

Bacterial lysates are rich in PAMPs, a diversity of evolutionarily conserved molecular structures that function as signatures of microbial presence. These components include lipoteichoic acids characteristic of Gram‐positive bacteria, peptidoglycans that form the structural skeleton of the bacterial wall, flagellin in bacterial flagella, and unmethylated CpG sequences in bacterial DNA. TLRs—particularly TLR2, TLR4, TLR5, and TLR9—expressed on the surface and in the endosomal compartments of DCs, macrophages, and epithelial cells, recognize these PAMPs and initiate complex signaling cascades that amplify the immune response [73].

NLRs complete this immunological surveillance network by sensing cytoplasmic fragments of bacterial peptidoglycan. Activation of NOD1 and NOD2 triggers signaling through nuclear factor kappa B (NF‐κB), a master transcription factor that orchestrates the expression of genes involved in inflammation, producing proinflammatory cytokines such as TNF‐α and interleukin‐6 (IL‐6), and priming the inflammasome for subsequent activation. This coordinated engagement of PRRs leads to DC maturation, upregulation of co‐stimulatory molecules essential for T lymphocyte activation (CD80, CD86), and efficient presentation of antigens to the adaptive immune system [74, 75].

### 7.2 Inflammasome Activation: Fine‐Tuning Inflammation

Multiple recent studies report that bacterial lysates modulate the activity of the NLRP3 inflammasome, a supramolecular multiprotein complex predominantly present in macrophages and intestinal epithelial cells, which functions as a sensor of cellular stress and danger signals. This modulation controls the secretion of interleukin‐1 beta (IL‐1β) and interleukin‐18 (IL‐18), potent proinflammatory cytokines that amplify the innate immune response and facilitate the transition to adaptive immunity [76, 77].

The regulation of inflammasome activity by lysates represents a sophisticated balancing act: it can enhance host defense against pathogens by increasing the production of proinflammatory cytokines when necessary, but at the same time prevents excessive inflammation that could generate significant tissue damage and contribute to the chronicity of pathological processes. This dual capacity for modulation—stimulation when protection is required, tempering when there is a risk of injury—explains the therapeutic versatility of lysates in various clinical contexts [78].

### 7.3 Polarization of T Helper Lymphocyte Subsets: Stimulation of Adaptive Immunity

Bacterial lysates can profoundly influence the balance between different T helper lymphocyte subpopulations (Th1, Th2, Th17, and Treg) depending on the specific disease context, creating immunological profiles tailored to particular therapeutic needs.

In infection models, lysates promote Th1 and Th17 responses, characterized by the production of interferon‐gamma (IFN‐γ) and interleukin‐17 (IL‐17), cytokines essential for efficient microbial clearance through macrophage activation and neutrophil recruitment. This coordinated proinflammatory profile maximizes the host's ability to eliminate invading pathogens [79, 80].

In contrast, in models of allergic asthma and atopic dermatitis, lysates suppress Th2 responses characterized by the production of IL‐4, IL‐5, and IL‐13, thereby reducing IgE production and tissue eosinophilia, two parameters of allergic inflammation. This shift from Th2 to more balanced or Th1‐dominant profiles is the basis for therapeutic efficacy in allergic diseases [81, 82].

In autoimmune disease models such as EAE and experimental colitis, the induction of regulatory T lymphocytes (Treg) characterized by the expression of the transcription factor Foxp3 is crucial for promoting immunological tolerance and reducing inflammatory tissue damage. Treg cells suppress excessive autoreactive responses through multiple mechanisms: secretion of immunosuppressive cytokines (IL‐10, TGF‐β), competitive consumption of IL‐2, and direct inhibition of DCs and effector T lymphocytes [83, 84].

### 7.4 Macrophage Polarization: Functional Versatility in Context

Macrophages, versatile phagocytic cells with remarkable functional plasticity, can be polarized by bacterial lysates toward the M1 (proinflammatory, tumoricidal) or M2 (anti‐inflammatory, pro‐reparative) phenotype depending on the desired immunological context and local microenvironmental signals.

In cancer models, M1 polarization of macrophages enhances antitumor cytotoxicity through the production of reactive oxygen and nitrogen species, the secretion of proinflammatory cytokines that activate cytotoxic T lymphocytes, and the direct presentation of tumor antigens to the adaptive immune system. This M1 orientation transforms macrophages from potential tumor accomplices to active allies of the antitumor response [85, 86].

In contrast, in experimental colitis models, M2 macrophage responses promote tissue repair and resolution of inflammation through the secretion of growth factors and anti‐inflammatory cytokines such as IL‐10, facilitating healing of the damaged intestinal mucosa and preventing chronic inflammation. This functional versatility illustrates how lysates can be strategically used to direct the immune response toward specific therapeutic targets [87, 88].

### 7.5 Trained Immunity: Epigenetic Memory of the Innate System

One of the most fascinating mechanisms recently elucidated is the ability of bacterial lysates to induce “trained immunity”—a long‐term functional reprogramming of innate immune cells (monocytes, macrophages, natural killer cells) that results in faster and stronger responses to secondary challenges, even with pathogens completely different from the initial stimulus. This phenomenon is mediated by epigenetic modifications—particularly DNA methylation and histone modifications—that alter the chromatin accessibility of genes involved in the immune response, allowing for faster and more robust gene expression upon restimulation [63, 89]. Trained immunity is a key mechanism explaining the prophylactic effect of lysates in recurrent respiratory infections, where initial exposure to the lysate “primes” the immune system to respond more effectively to subsequent infections. Emerging evidence suggests that this mechanism may also operate in models of intestinal and urinary tract infections and even in systemic protection against various inflammatory pathologies [90, 91].

### 7.6 The Gut–Brain Axis and Systemic Immunity: Remote Connectivity

Oral administration of bacterial lysates modulates the composition of the gut microbiota, generating changes in the taxonomic and metabolic profile of the commensal microbial community. These microbial exchanges, in turn, influence systemic immune tone and inflammation in the central nervous system through a complex communication network known as the gut–brain axis [61, 92].

In EAE models of experimental multiple sclerosis, lysate‐induced changes in the microbiota promote the expansion of the regulatory T lymphocyte population and reduce neuroinflammation, highlighting the crucial importance of the tripartite microbiota–immune system–central nervous system dialogue. This observation opens fascinating therapeutic perspectives for inflammatory neurological diseases through indirect modulation, mediated by the gut microbiota [60, 67].

Mechanisms of this long‐distance communication include: the production of microbial metabolites (short‐chain fatty acids, tryptophan and its derivatives) that cross the blood–brain barrier and directly influence glial and neuronal function; signaling through the vagus nerve, which transmits information about intestinal status to brain structures; and modulation of circulating cytokines that affect blood–brain barrier permeability and activation of microglia, the brain's resident macrophages [93, 94].

## 8 Translational Potential and Limitations of Bacterial Lysates: From the Lab Bench to the Patient Bedside

Bacterial lysates have demonstrated robust and consistent immunomodulatory effects in various murine disease models, accumulating an impressive body of preclinical evidence supporting their therapeutic potential. However, the translation of these experimental findings into effective and safe clinical applications requires careful consideration of the multiple biological, methodological, and regulatory factors that may influence the successful transfer from animal models to human patients. This transition from basic research to clinical practice represents one of the greatest challenges in the development of new therapies, requiring a systematic and critical approach (Table 3).

**Table 3:** Advantages and limitations of bacterial lysates in murine preclinical research.

Advantages	Limitations
Increased safety compared to live probiotics (no risk of bacteremia or transfer of resistance genes)	Lack of standardization of preparation protocols and dosages across studies
Simultaneous modulation of innate and adaptive immunity	Predominance of preventive study designs (few therapeutic studies conducted after disease onset)
Broad applicability across multiple disease models (respiratory, allergic, intestinal, autoimmune, oncological)	Significant differences between the murine and human immune systems
Potential for use as adjuvants in immunotherapy (e.g., anti‐CTLA‐4)	Limited data on long‐term safety and efficacy
Stability and ease of administration (especially oral formulations)	High heterogeneity of preparations (commercial vs. research‐grade), which hinders comparability
Ability to induce trained immunity	Few studies in humanized models or species more closely related to humans

### 8.1 Translational Potential: Promising Premises for Clinical Applications

Preclinical data provide substantial support for the use of bacterial lysates as prophylactic or adjuvant agents designed to modulate immune responses in the broad spectrum of pathologies investigated: recurrent respiratory and nonrespiratory infections, autoimmune diseases with a chronic inflammatory component, allergic and atopic diseases, and even cancer in the context of oncological immunotherapy. This therapeutic versatility derives from the ability of lysates to simultaneously and coordinately engage multiple immunological pathways, creating immunomodulatory profiles adaptable to the specific requirements of each pathology [56, 57, 95].

Unlike live probiotics, which contain metabolically active and replicable microorganisms, bacterial lysates are nonreplicative preparations composed of thermally or mechanically inactivated bacteria and their structural components. This fundamental feature dramatically reduces the risks associated with bacteremia (invasion of the blood by viable bacteria), bacterial translocation from the gastrointestinal tract to other anatomical compartments, or horizontal transfer of antibiotic resistance genes between bacteria. This increased safety makes lysates particularly suitable for vulnerable populations such as immunocompromised patients, frail elderly with multiple comorbidities, or infants with immature immune systems [96, 97].

The demonstrated ability to simultaneously engage multiple immunological pathways—innate immunity via PRRs, adaptive immunity via T‐lymphocyte polarization, and immunity trained through epigenetic reprogramming of innate immune cells—gives lysates a remarkably broad therapeutic applicability. This versatility allows their use in extremely diverse clinical contexts, from local mucosal infections to systemic immune modulation in autoimmune or neoplastic diseases, making them ideal candidates for multi‐indication therapeutic platforms [63, 98].

Emerging evidence from murine tumor models suggests a significant synergistic potential between bacterial lysates and immune checkpoint inhibitors, such as anti‐CTLA‐4 or anti‐PD‐1/PD‐L1 antibodies. This synergy is based on the ability of lysates to remodel the tumor microenvironment, deplete immune suppressor cells (intratumoral Treg, M2 macrophages), and amplify cytotoxic responses mediated by CD8+ T lymphocytes. These observations open a promising avenue for the development of combinatorial therapies in oncology, where microbiome modulation and innate immunity engagement could potentiate the efficacy of conventional immunotherapy [99, 100].

### 8.2 Major Limitations: Challenges in Clinical Translation

One of the most significant limitations in mouse‐to‐human translation is the fundamental differences between murine and human immune responses, as well as the composition and functionality of the microbiota of the two species. Laboratory mice are raised under conditions of relative sterility with minimal microbial exposure compared to humans, have a much faster metabolic rate, exhibit different proportions of immune subpopulations, and respond differently to inflammatory stimuli. These fundamental biological discrepancies limit the direct predictability of efficacy and safety observed in murine models for human applications [13, 101, 102].

Bacterial lysates investigated in the scientific literature vary considerably in multiple critical dimensions: the component bacterial strains (mono‐species vs*.* multi‐species, Gram‐positive vs. Gram‐negative bacteria), the preparation methods (mechanical lysis, heat inactivation, sonication, chemical treatment), the concentrations and doses administered, and the routes of administration used (oral, intranasal, systemic). This extensive methodological heterogeneity significantly complicates the standardization of preparations, the reproducibility of results between laboratories, and the direct comparability of studies, representing a major barrier to the development of consistent pharmaceutical products and regulatory approval [103, 104].

Most preclinical studies focus on the prophylactic administration of lysates, where treatment precedes the induction of experimental disease, allowing the immune system to be “primed” before pathological challenge. In contrast, the therapeutic efficacy of lysates after the onset of clinical manifestations—the scenario most relevant to real‐world clinical practice—remains underexplored. This gap is critical because the mechanisms involved in disease prevention may differ substantially from those required to reverse established pathological processes, and the response to treatment may be significantly different in tissues already remodeled by inflammation [105, 106].

Although key mechanisms such as engagement of PRRs, induction of regulatory T lymphocytes (Treg), and trained immunity have been identified and partially characterized, the full spectrum of cellular and molecular effects of bacterial lysates is not yet fully elucidated. Fundamental questions remain unanswered: What are all the activated cell types and their mechanisms of action? How do lysates interact with the resident microbiota and what are the consequences of this interaction? What are the precise epigenetic changes that mediate trained immunity? How do responses vary depending on the host genetic background and baseline immunological status? This incomplete mechanistic understanding limits our ability to predict efficacy, optimize treatment protocols, and identify predictive biomarkers of response [64, 98].

Most preclinical studies are of relatively short duration, encompassing a few weeks to a few months of treatment and follow‐up. The effects of chronic and long‐term administration of bacterial lysates—including the impact on the stability and diversity of the gut microbiota, the consequences on immunological memory and tolerance, the potential for desensitization or tolerance to repeated stimuli, and the risks of late adverse effects—require extensive further investigation. This gap in long‐term safety data is particularly relevant for indications that would require chronic administration, such as the prevention of recurrent infections or the management of persistent allergic diseases [107].

### 8.3 Strategies for Optimizing Translation: Building Bridges Between Research and the Clinic

The development of rigorous and reproducible manufacturing protocols is an absolute priority. This includes: the selection and complete genetic characterization of the bacterial strains used, with documentation of their virulence and immunogenicity profile; standardization of inactivation methods to ensure consistency of active components and complete absence of viable bacteria; validation and quality control by sophisticated analytical methods (mass spectrometry, chromatography, immunoassays); and establishment of pharmacokinetic and pharmacodynamic parameters to guide dosing regimens [108].

The use of humanized mouse models—animals with partially reconstituted immune systems with human cells—or higher animals such as pigs or nonhuman primates, which have greater physiological and immunological similarities to humans, can significantly improve the translational relevance of preclinical data. These advanced models allow testing of human immune responses in the context of a whole organism, providing more reliable predictions of clinical efficacy and safety [109, 110].

Systematic incorporation of microbiome analyses (metagenomics, metatranscriptomics, metabolomics) and detailed immunophenotyping (multiparametric flow cytometry, single‐cell transcriptomics, epigenetic profiling) into preclinical and clinical studies can guide biologically based patient stratification and the identification of biomarkers predictive of response. This precision medicine approach allows for the personalization of therapy according to the individual immunological and microbial profile of each patient, maximizing efficacy and minimizing adverse responses [111, 112].

Initial clinical trials should focus primarily on rigorous assessment of safety and tolerability at escalating doses, characterization of pharmacodynamics by measuring immune biomarkers (cytokines, cellular phenotypes, antibodies), monitoring of effects on the gut microbiota by longitudinal metagenomic sequencing, and preliminary identification of clinical efficacy signals. This pragmatic proof‐of‐concept design can rapidly validate therapeutic potential and inform the design of later‐phase pivotal trials [108, 113].

## 9 Conclusions

Murine studies provide a compelling and multifaceted proof of concept for the use of bacterial lysates as versatile immunomodulatory agents. Beneficial effects have been demonstrated across a broad spectrum of inflammatory, infectious, allergic, autoimmune, and neoplastic pathologies. Among these, the evidence is most consistent and translationally advanced in models of respiratory tract infections—the original and still most established clinical indication of bacterial lysates—where both oral and intranasal administration of standardized preparations such as OM‐85 have shown protection against viral and bacterial challenges, including superinfection.

Promising results have also been obtained in allergic asthma, recurrent urinary tract infections, experimental colitis, neuroinflammation, and as adjuvants in cancer immunotherapy. Nevertheless, the full realization of the therapeutic potential of bacterial lysates requires several critical steps: the development of standardized manufacturing and quality‐control protocols, a shift toward therapeutic (rather than exclusively preventive) experimental designs, deeper mechanistic elucidation, and rigorous bridging studies in more human‐relevant models.

Only through such a methodical and translationally oriented approach will it be possible to convert the substantial preclinical promise of bacterial lysates into effective and safe clinical applications for patients.

## Author Contributions

**Diana‐Larisa Ancuța:** conceptualization, investigation, methodology, writing – original draft, formal analysis, data curation, project administration, funding acquisition, software, resources. **Cristin Coman:** conceptualization, investigation, writing – review and editing, visualization, validation, methodology, formal analysis, supervision. All authors critically reviewed the manuscript, approved the final version, and agree to be responsible for the accuracy and integrity of the work.

## Conflicts of Interest

The authors declare no conflicts of interest.

## Supporting information


Supporting File

## Data Availability

The data that support the findings of this study are available from the corresponding author upon reasonable request.
