# Supplementary material for: Preclinical Evaluation of Bacterial Lysates in Murine Disease Models: A Systematic Review
Source: Immun Inflamm Dis. 2026 Oct 15;14(10):e70553. doi: 10.1002/iid3.70553 (PMC13670408; doi:10.1002/iid3.70553)
Supplement: Supplementary file 1 [file IID3-14-e70553-s001.docx]

| SUPPLEMENTARY TABLE 1 | | | | | | | | | | | |
| --- | --- | --- | --- | --- | --- | --- | --- | --- | --- | --- | --- |
| Id | Year | Mouse strain | Lysate composition | Inactivation method | Disease model | Route | Design | Main findings short | Category | Commercial/reasearch | DOI |
| 1 | 2024 | BALB/c, C57BL/6, CD-1 | OM-85 BV (polybacterial) | Thermal / tyndallized | Influenza + Salmonella infection | Oral (gavage) | Preventive | Protected against influenza and Salmonella; reduced morbidity | Respiratory infection | Commercial (OM-85) | 10.1016/j.intimp.2010.06.009 |
| 2 | 2025 | BALB/c | OM-85 | Thermal | Influenza followed by *S. pneumoniae* superinfection | Oral (gavage) | Preventive | Primary antiviral stimulation reduced susceptibility to secondary bacterial infection; improved survival | Respiratory infection | Commercial (OM-85) | 10.1186/s13052-018-0569-7 |
| 3 | 2023 | Mice | Polyvalent bacterial lysate (PABL/PMBL) | Mechanical / Chemical | Pneumococcal pneumonia | Intranasal | Preventive (single dose) | Single i.n. dose protects against lethal S. pneumoniae; MyD88-dependent; independent of neutrophils/IL-17A/caspase-1 | Respiratory infection | Research-grade | 10.3389/fimmu.2021.562244 |
| 4 | 2024 | Mice | OM-85 | Thermal | OVA-induced asthma | Oral | Preventive | Recruitment of regulatory T cells to airways; prevents asthma | Asthma / Allergy | Commercial (OM-85) | 10.1038/mi.2010.51 |
| 5 | 2019 | Mice | OM-85 | Thermal | OVA asthma model | Oral | Therapeutic/Preventive | Reduced airway inflammation, eosinophilia, Th2 cytokines | Asthma / Allergy | Commercial (OM-85) | 10.1007/s00408-017-0003-8 |
| 6 | 2024 | Various (incl. GF) | OM-85 | Thermal | Experimental asthma (OVA, Alternaria) | Intranasal | Preventive | Blocks asthma by targeting DCs and epithelium/IL-33/ILC2 axis; lower cumulative dose than oral | Asthma / Allergy | Commercial (OM-85) | 10.1016/j.jaci.2021.09.013 |
| 7 | 2024 | BALB/c GF & Ex-GF | OM-85 | Thermal | Allergen-induced lung eosinophilia | Intranasal | Preventive | Airway lysate sufficient to inhibit eosinophilia even in germ-free mice | Asthma / Allergy | Commercial (OM-85) | 10.1093/jleuko/qiae047 |
| 8 | 2021 | BALB/c | OM-85 Broncho-Vaxom | Thermal | Asthma + TiO2 pollutant | Oral | Preventive/Therapeutic | Impact on lung microbiota composition in asthma model with pollutant | Asthma / Allergy | Commercial (OM-85) | 10.1093/lambio/ovaf089 |
| 9 | 2022 | BALB/c | *Lactococcus lactis* (For t 2) - sonicated & autoclaved groups | Sonicated / Heat-inactivated | Forcipomyia taiwana allergy | Gavage | Immunotherapy | Includes lysate and heat-inactivated groups vs viable | Asthma / Allergy | Research-grade | 10.1016/j.waojou.2025.101073 |
| 10 | 2014 | BALB/c, SCID | *Lactobacillus casei* DN-114 001 lysate | Thermal / mechanical | DSS-induced colitis | Oral | Preventive | Ameliorates colitis by strengthening gut barrier; effect requires intact immune system | IBD / Colitis | Research-grade | 10.1371/journal.pone.0027961 |
| 11 | 2015 | Mice | *Lactococcus petauri* GB97 lysate | Thermal (heat-killed) | DSS colitis / intestinal inflammation | Oral | Preventive/Therapeutic | Effects on inflammation, barrier function, gut microbiota | IBD / Colitis | Research-grade | 10.1128/spectrum.01334-23 |
| 12 | 2022 | BALB/c | UNAM-HIMFG polyvalent bacterial lysate | Not fully specified | Uropathogenic *E. coli* CFT073 UTI | Sublingual | Therapeutic | Faster urinary clearance, absence of chronic inflammation, balanced cytokine response | UTI | Research-grade / institutional | 10.3390/ijms25189876 |
| 13 | 2023 | BALB/c | Polyvalent (*E.coli + Klebsiella, Citrobacter, Proteus, Staph, Enterococcus*) | Not fully specified | Recurrent UTI | Oral | Prophylactic / Therapeutic | Accelerated bacteriuria clearance, reduced bladder/kidney inflammation, long-term sterility 50-80% | UTI | Research-grade / institutional | 10.3390/ijms25116157 |
| 14 | 2022 | Mice | Heat-killed multi-strain probiotics (postbiotic) | Thermal | Antibiotic-resistant primary & recurrent UTI | Oral | Prophylactic | Durable protection against recurrent UTI | UTI | Research-grade | 10.3390/antibiotics14070634 |
| 15 | 2021 | Mice | Viable and heat-inactivated probiotic strains | Thermal | Murine UTI | Oral | Therapeutic | Therapeutic effects of inactivated strains against UTI | UTI | Research-grade | 10.38212/2224-6614.3474 |
| 16 | 2025 | C57BL/6 | *Parabacteroides distasonis* lysate | Thermal / mechanical | Experimental autoimmune encephalomyelitis (EAE) | Oral | Preventive | Delayed onset, reduced severity; Treg expansion in LN, microbiota remodeling | Neuroinflammation (EAE) | Research-grade | 10.3389/fimmu.2024.1475126 |
| 17 | 2017 | BALB/c | *Lactobacillus acidophilus* lysate + anti-CTLA-4 | Thermal | AOM/DSS colorectal cancer | Intragastric | Combined therapeutic | Reduced tumor burden; increased CD8+ T cells, decreased Treg and M2 macrophages | Cancer | Research-grade | 10.1038/s41598-019-56661-y |
| 18 | 2025 | C57Bl/6 | OM-85 | Thermal | High-fat diet obesity, insulin resistance, NASH | Gavage | Preventive/Therapeutic | Attenuates obesity, IR, gut dysbiosis and NASH | Metabolic / Other | Commercial (OM-85) | 10.1016/j.biopha.2024.117710 |
| 19 | 2023 | C57BL/6J | OM-85 (Broncho-Vaxom) | Thermal | LPS-induced acute lung injury | Gavage | Preventive/Therapeutic | Attenuates inflammation in ALI model | Acute lung injury / Fibrosis | Commercial (OM-85) | 10.3390/ijms25137135 |
| 20 | 2025 | Mice | Heat-killed Lactobacilli preparations | Thermal | Experimental cutaneous wounds | Topical / other | Therapeutic | Promote healing of cutaneous wounds | Metabolic / Other | Research-grade | 10.3390/cells10113264 |
| 21 | 2025 | BALB/c | OM-85 | Thermal | RSV infection | Intranasal | Protective | Airway OM-85 protects against RSV; inhibits viral replication, reduces inflammation | Respiratory infection | Commercial (OM-85) | 10.3389/fimmu.2022.867022 |
| 22 | 2025 | Mice | OM-85 Broncho-Vaxom | Thermal | OVA-induced allergic rhinitis | Oral | Preventive (long-term) | Long-term pretreatment suppressed nasal symptoms, eosinophils, Th2 cytokines, IgE/IgG1 | Asthma / Allergy | Commercial (OM-85) | 10.2500/ajra.2013.27.4021 |
| 23 | 2024 | BALB/c | OM-85 BV | Thermal | Aspergillus fumigatus-induced chronic rhinosinusitis | Intranasal | Therapeutic (adjunct) | Reduced SOCS1/SOCS3, TNF-alpha, IFN-gamma; comparable benefit to amoxicillin | Respiratory infection | Commercial (OM-85) | PMID: 26261565 |
| 24 | 2025 | Mice | Polyvalent bacterial lysate (aerosolized) | Thermal | Bleomycin-induced pulmonary fibrosis | Aerosolized | Preventive | Reduced fibrotic score and hydroxyproline; decreased IL-4, increased IFN-gamma/IL-4 ratio | Acute lung injury / Fibrosis | Commercial-like | 10.1007/s10753-022-01653-w |
| 25 | 2023 | BALB/c | OM-85 (Broncho-Vaxom) | Thermal | OVA-induced asthma | Oral | Preventive | Suppressed AHR, IgE and pulmonary inflammation via enhanced Treg and SCFA (acetate) | Asthma / Allergy | Commercial (OM-85) | 10.1111/pai.14018 |
| 26 | 2024 | BALB/c, C57BL/6 (Myd88-/-Trif-/-) | OM-85 | Thermal | SARS-CoV-2 receptor expression (Ace2/Tmprss2) | Intranasal | Preventive/Mechanistic | Downregulated Ace2/Tmprss2 in mouse lung (Myd88/Trif-dependent); inhibited S1 binding | Respiratory infection | Commercial (OM-85) | 10.1016/j.jaci.2021.11.019 |
| 27 | 2019 | BALB/c | OM-85 | Thermal | Neonatal rhinovirus infection (maternal treatment) | Oral (maternal, gestation) | Preventive (prenatal) | Maternal OM-85 enhanced offspring protection against neonatal rhinovirus | Respiratory infection | Commercial (OM-85) | 10.1002/cti2.1303 |
| 28 | 2024 | C57BL/6 | OM-85 | Thermal | OVA allergic airway inflammation in adult offspring (ILC2) | Oral (maternal, gestation/lactation) | Preventive (prenatal) | Maternal OM-85 reduced offspring ILC2-driven allergy via IL-33/ILC2 and SCFA | Asthma / Allergy | Commercial (OM-85) | 10.1111/pai.70044 |
| 29 | 2024 | Mice | OM-85 | Thermal (alkaline lysis) | Mechanistic (inflammasome + IFN-beta) | *In vitro* + *in vivo* context | Mechanistic | OM-85 modulates interferon-beta production and inflammasome activity | Respiratory infection | Commercial (OM-85) | 10.1038/srep43844 |
| 30 | 2021 | C57BL/6J | Heat-killed *Lactococcus lactis* subsp. lactis L8 | Thermal (heat-killed) | DSS-induced colitis | Oral | Preventive/Therapeutic | Alleviated clinical score and histological abnormalities; reduced colon TNF-alpha | IBD / Colitis | Research-grade | 10.3164/jcbn.24-194 |
| 31 | 2022 | BALB/c | Heat-killed *Bifidobacterium longum* BBMN68 (in pasteurized yogurt) | Thermal (heat-killed) | Mugwort pollen-induced allergic airway responses | Oral | Preventive | Lower IgE, decreased Th2 cytokines, alleviated airway inflammation; modulated gut microbiota | Asthma / Allergy | Research-grade | 10.3390/foods12102049 |
| 32 | 2014 | C57 mice (female) | OM-85 | Thermal | Bleomycin-induced pulmonary fibrosis | Aerosol | Therapeutic | Mitigated fibrosis; inhibited Notch1/Hes1; modulated IFN-gamma/IL-4 ratio | Acute lung injury / Fibrosis | Commercial (OM-85) | 10.1038/s41598-025-89874-5 |
| 33 | 2015 | Mice | Heat-killed Lacticaseibacillus paracasei 6235 | Thermal (heat-killed) | DSS-induced colitis | Oral | Preventive/Therapeutic | More effective than live form; modulated microbiota and MAPK/NF-kB pathways | IBD / Colitis | Research-grade | 10.1039/D4FO04873C |
| 34 | 2022 | Mice | Heat-killed multi-strain postbiotic | Thermal | Primary & recurrent antibiotic-resistant UTI | Oral | Prophylactic | Durable protection; high sterile urine rates post-treatment | UTI | Research-grade | 10.3390/antibiotics14070634 |
